# Differences in transcription between free-living and CO_2_-activated third-stage larvae of *Haemonchus contortus*

**DOI:** 10.1186/1471-2164-11-266

**Published:** 2010-04-27

**Authors:** Cinzia Cantacessi, Bronwyn E Campbell, Neil D Young, Aaron R Jex, Ross S Hall, Paul JA Presidente, Jodi L Zawadzki, Weiwei Zhong, Boanerges Aleman-Meza, Alex Loukas, Paul W Sternberg, Robin B Gasser

**Affiliations:** 1 Department of Veterinary Science, The University of Melbourne, Werribee, Victoria, Australia; 2 Department of Primary Industries, Attwood, Victoria, Australia; 3 Department of Biochemistry and Cell Biology, Rice University, Houston, Texas, USA; 4 Department of Information Technology, Polytechnic University of Victoria, Mexico; 5 James Cook University, Cairns, Queensland, Australia; 6 Biology Division, California Institute of Technology, Pasadena, California, USA

## Abstract

**Background:**

The disease caused by *Haemonchus contortus*, a blood-feeding nematode of small ruminants, is of major economic importance worldwide. The infective third-stage larva (L3) of this gastric nematode is enclosed in a cuticle (sheath) and, once ingested with herbage by the host, undergoes an exsheathment process that marks the transition from the free-living (L3) to the parasitic (xL3) stage. This study explored changes in gene transcription associated with this transition and predicted, based on comparative analysis, functional roles for key transcripts in the metabolic pathways linked to larval development.

**Results:**

Totals of 101,305 (L3) and 105,553 (xL3) expressed sequence tags (ESTs) were determined using 454 sequencing technology, and then assembled and annotated; the most abundant transcripts encoded transthyretin-like, calcium-binding EF-hand, NAD(P)-binding and nucleotide-binding proteins as well as homologues of *Ancylostoma*-secreted proteins (ASPs). Using an *in silico*-subtractive analysis, 560 and 685 sequences were shown to be uniquely represented in the L3 and xL3 stages, respectively; the transcripts encoded ribosomal proteins, collagens and elongation factors (in L3), and mainly peptidases and other enzymes of amino acid catabolism (in xL3). *Caenorhabditis elegans *orthologues of transcripts that were uniquely transcribed in each L3 and xL3 were predicted to interact with a total of 535 other genes, all of which were involved in embryonic development.

**Conclusion:**

The present study indicated that some key transcriptional alterations taking place during the transition from the L3 to the xL3 stage of *H. contortus *involve genes predicted to be linked to the development of neuronal tissue (L3 and xL3), formation of the cuticle (L3) and digestion of host haemoglobin (xL3). Future efforts using next-generation sequencing and bioinformatic technologies should provide the efficiency and depth of coverage required for the determination of the complete transcriptomes of different developmental stages and/or tissues of *H. contortus *as well as the genome of this important parasitic nematode. Such advances should lead to a significantly improved understanding of the molecular biology of *H. contortus *and, from an applied perspective, to novel methods of intervention.

## Background

Parasitic nematodes of livestock animals are of major socio-economic importance worldwide due to the diseases and production losses that they cause. *Haemonchus contortus *(order Strongylida) is one of the most important nematodes of the abomasum (stomach) of small ruminants. The disease (= haemonchosis) caused by this parasite represents ~15% of all gastrointestinal diseases of small ruminants worldwide and causes major financial losses http://www.fao.org. *Haemonchus contortus *is a blood-feeding worm that causes anaemia and associated complications, leading to death in severely affected animals [1]. In the abomasum of the ruminant host, the oviparous adult females release eggs *via *the faeces into the environment, in which first-stage larvae (L1s) develop and then hatch (within ~1 day, depending on conditions). The L1s develop into second-stage larvae (L2s), which then moult to become infective third-stage larvae (L3s). The cuticle from the L2 is retained as a sheath around the L3 and protects this stage from environmental pressures. Infective L3s are ingested with herbage by the host, pass through the forestomachs and undergo an exsheathment process to then establish, *via *the parasitic fourth-stage larvae (L4s), to dioecious haematophagous adults within ~3 weeks [2].

The exsheathment process marks the transition from the free-living (L3) to the parasitic (xL3) larval stage, at which *H. contortus *grows, develops and commences feeding on the blood of the host [1]. Early studies examined aspects of this process in nematodes in response to a host stimulus. Evidence indicated that at least two separate pathways control these responses [3-5]. These pathways are stimulated by gaseous CO_2 _and mediated by carbonic anhydrase, leading to the secretion of the neurotransmitter noradrenaline [3]. Noradrenaline then mediates the activation of genes required for further development and the release of exsheathment fluid to induce moulting of the L2 cuticle through a separate pathway. In the absence of CO_2 _stimulation, both pathways appear to be inhibited in L3s by an analogue of the "insect juvenile hormone", which is implicated in the regulation of development in insects [3-6]. It has been suggested that the CO_2 _cue is sensed by chemosensory neurons of the amphids [7,8], which are sensory organs located in the head of L3s and are exposed to the external environment *via *a pore [9]. The exsheathment process can be induced *in vitro *[10] and is recognised to suitably represent the process *in vivo *in the host animal [10,11]. In spite of knowledge of the exsheathment process, the role of carbonic anhydrase and the chemoreception are poorly understood in *H. contortus*, and the exact nature of the regulation and components of the "downstream" molecular pathways are not yet known [1].

Despite these substantial knowledge gaps for *H. contortus*, Rogers and Petronijevic [4] did hypothesize early that there is likely to be a specific pattern of gene expression during the development of nematodes, whereby genes which control continuous "house-keeping" processes and maintain survival are expressed on a constitutive basis, whereas genes which have specific functions in one or more stages of development are regulated for expression exclusively at specific developmental time points. Although studies of *H. contortus *have shown that the pattern of transcription differs between free-living and parasitic stages [12-14], there has been no detailed study of the molecular alterations occurring during the early phase of transition to parasitism in this parasite, although there is some information for the canine hookworm, *Ancylostoma caninum *[15-18]. Insights into the transcriptome of *H. contortus *during this critical phase of establishment in its host would enhance knowledge of developmental processes at the molecular level and might identify new intervention targets.

Advances in genomic, proteomic and bioinformatic technologies [19-22] now provide opportunities for exploring the molecular basis of developmental switches in *H. contortus *and other nematodes. In particular, the combined use of next-generation sequencing, such as 454 technology, SOLiD and Illumina/Solexa [19,23-25] and improved bioinformatic algorithms for the analysis and annotation of expressed sequence tag (EST) datasets [26] are suited to elucidate molecular changes at the transcriptomic level. In the present study, we (i) provide the first detailed insights into the transcriptome of *H. contortus *during its transition from the L3 to the exsheathed, parasitic xL3 stage using a next generation sequencing-based approach, (ii) predict, employing a number of bioinformatic approaches, the functional roles of these molecules in larval development and the metabolic pathways linked to this transition, and (iii) discuss the implications of the findings in relation to the fundamental, developmental biology of nematodes as well as applied aspects of developing new methods of nematode control.

## Results

**Figure 1:**
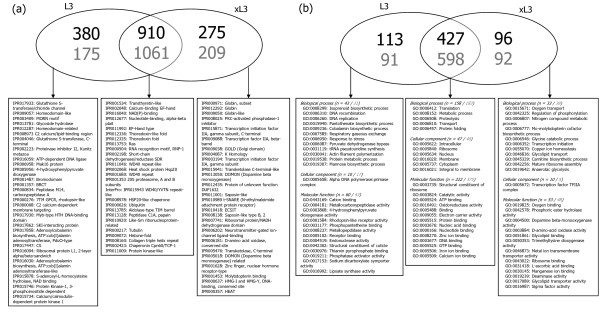
**Annotation**. Venn diagram displaying the number of protein domains (InterPro) (a) and Gene Ontology (GO) terms (b) common and uniquely represented in expressed sequence tag (ESTs; numbers in black) and genome sequence survey (GSS; in dark grey) data for the ensheathed (= L3) and exsheathed (= xL3) third larval stage of *Haemonchus contortus*. Descriptions of the 25 most abundant domains (a) and GO terms (b) are given in the boxes.

Totals of 101,305 (L3) and 105,553 (xL3) expressed sequence tags (ESTs; average of 210-216 bases in length) were generated using 454 technology. The vast majority (95%) of these ESTs matched publicly available genomic DNA sequences of *H. contortus*. A summary of the characteristics of the raw and assembled data is given in Table 1. For L3, 9,046 proteins were inferred from a total of 20,066 assembled nucleotide sequences (Table 2), of which 3,066 matched known proteins with 1,465 different domains (Figure 1a; Additional file 1). For xL3, 9,001 proteins were inferred from a total of 20,116 assembled nucleotide sequences (Table 2), of which 2,885 mapped to known proteins with 1,394 distinct domains (Figure 1a; Additional file 1). For both L3 and xL3, the 'transthyretin-like' (8%, IPR001534), 'calcium-binding EF-hand' (6.7%, IPR002048), 'nicotinamide adenine dinucleotide(phosphate) [NAD(P)]-binding domain' [NAD(P)-binding] (5%, IPR016040), and 'nucleotide-binding, alpha beta plait' (5%, IPR012677) domains were most commonly detected (see Figure 1a; Additional file 1).

**Table 1:** Characteristics of the expressed sequence tags (ESTs).

Numbers of	L3	xL3
Sequences before assembly of ESTs (average length)	101305	105553
Sequences after assembly	11824	11671
Contigs (average length ± standard deviation)	6299 (352.7 bp ± 91.9)	5574 (361.6 bp ± 99.8)
Singletons (average length ± standard deviation)	5525 (274.1 bp ± 45.7)	6097 (278.7 bp ± 60.2)
Clusters matching genomic sequences	10823	11021
Total number of unique clusters (ESTs + GSS)	20066	20116

Numbers of ESTs determined from cDNA libraries representing the ensheathed (= L3) or exsheathed (= xL3) third larval stage of *Haemonchus contortus*, and numbers of sequences (including contigs and singletons) and clusters and their lengths (in brackets) before and after assembly.

**Table 2:** Results of the bioinformatic analyses

	Numbers of ESTs/genomic sequences in L3	Numbers of ESTs/genomic sequences in xL3
Sequences with ORFs	6847/2199	6696/2305
InterPro	1876/1190	1614/1271
GO	1382/505	1171/540
KOBAS (pathway mapping)	2144/688	1788/1021
*C. elegans *homologues/orthologues	2975/2630	2922/2782
Homologues in organisms other than nematodes	2069/928	1689/1023
No known homologues in available databases	3274/4968	3349/4709

Results of the bioinformatic analyses of expressed sequence tag (EST) and genomic sequences representing the ensheathed (= L3) or exsheathed (= xL3) third larval stage of *Haemonchus contortus*. Numbers of sequences with open reading frames (ORFs) and of predicted proteins classified according to their domains (InterPro), Gene Ontologies (GO) and/or biological pathways (KOBAS). The numbers of sequences with orthologues or homologues in *Caenorhabditis elegans*, other parasitic nematodes and/or organisms other than nematodes are also listed.

The subsequent annotation of the proteins inferred for the L3 stage revealed 1,229 different Gene Ontology (GO) terms, of which 334 were linked to 'biological process', 116 'cellular component' and 779 'molecular function' (see Figure 1b; Additional file 2). For xL3, 1,213 GO terms were identified, of which 330 represented 'biological process', 93 'cellular component' and 790 'molecular function'. For both L3 and xL3, the most common GO terms were 'translation' (9.2%, GO:0006412) and 'metabolic process' (7.1%, GO:0008152) for 'biological process', 'intracellular' (18.7%, GO:0005622) and 'ribosome' (15.1%, GO:0005840) for 'cellular component' and 'structural constituent of ribosome' (9%, GO:0003735) and 'catalytic activity' (6.3%, GO:0003824) for 'molecular function' (see Figure 1b; Additional file 2). Pathway mapping using KEGG Orthology-Based Annotation System (KOBAS) predicted a total of 253 different pathways, of which the most represented were 'ribosome', 'oxidative phosphorylation', 'chaperones and folding catalysts' (see Additional file 3).

Subtractive bioinformatic analysis identified 560 and 685 nucleotide sequences as being uniquely transcribed in the L3 and xL3 stages, respectively [available *via *http://research.vet.unimelb.edu.au/gasserlab/index.html]. The uniqueness of selected (n = 30) transcripts was verified by independent reverse transcription (rt)PCR-coupled sequencing of amplicons. Of the sequences unique to L3, 336 had orthologues in *Caenorhabditis elegans *(Additional file 4), 15.7% of which encoded ribosomal proteins (n = 32), collagens and elongation factors (n = 21). Of the 555 functional domains predicted for all proteins, 'glutathione S-transferase/chloride channel' (4.7%, IPR017933), 'homeodomain-like' (3.7%, IPR009057) and 'MORN motif' (3%, IPR003409, Figure 1a; Additional file 1) were abundant. Among the 204 GO terms identified in the L3-'unique' dataset, the most common were 'isoprenoid biosynthetic process' (4.6%, GO:0008299) 'DNA recombination' (3%, GO:0006310) and 'DNA replication' for 'biological process'; 'alpha DNA polymerase, primase complex' (1.1%, GO:0005658) for 'cellular component'; and, 'cation binding' (6.8%, GO:0443169), 'metallo carboxypeptidase activity' (5%, GO:0004181) and '4-hydroxyphenylpyruvate dioxygenase activity' (1.8%, GO:0003868) for 'molecular function'(Figure 1b; Additional file 2). KOBAS mapping revealed links to 21 different pathways, of which 'biosynthesis and biodegradation of secondary metabolites' and 'cell motility and secretion' were the most commonly represented (Additional file 3).

Of the 685 sequences unique to xL3, 421 had known orthologues in *C. elegans*, the largest number (i.e. 5%) representing peptidases and other enzymes of the amino acid catabolism (n = 21) (Additional file 4). Among the 484 functional domains identified in the InterProScan analysis, 'globin' or 'globin-like' domains (19.7%, IPR000971, IPR012292 and IPR009050) had the greatest representation (Figure 1a; Additional file 1). The GO analysis identified a total of 188 terms, the most common being 'oxygen transport' (16.9%, GO:0015671), 'regulation of phosphorylation' (9.1%, GO:0042325) and 'nitrogen compound metabolic process' (4.7%, GO:0006807) for 'metabolic process'; 'transcription factor TFIIA complex' (10.3%, GO:0005672) for 'cellular component'; and, 'oxygen binding' (11%, GO:0019825), 'phosphoric ester hydrolase activity' (6.3%, GO:0042578) and 'dopamine beta-monooxygenase activity' (2.6%, GO:0004500) for 'molecular function' (Figure 1b; Additional file 2). Among the seven KOBAS pathways predicted for the xL3-specific data, 'cytokine-cytokine receptor interaction' and 'D-arginine and D-ornithine metabolism' were most commonly represented (Additional file 3).

**Figure 2:**
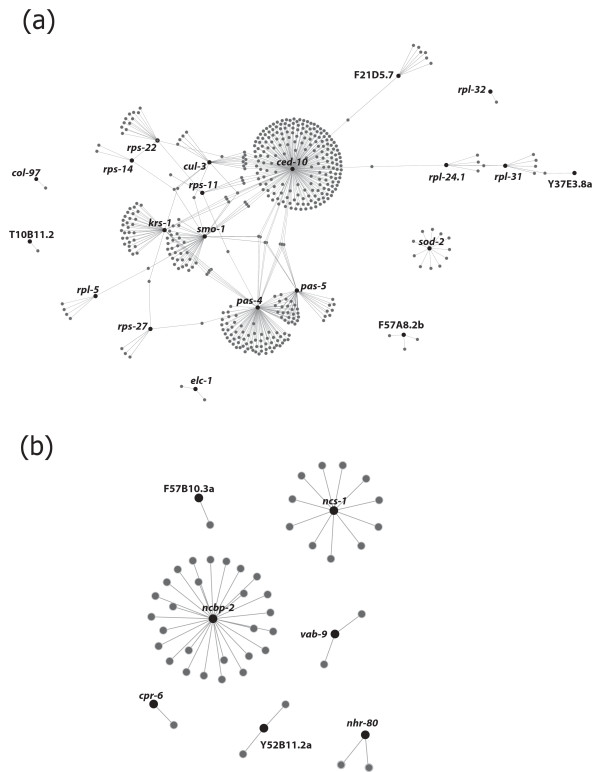
**Probabilistic genetic interaction networking**. Genetic interaction networks predicted for *Caenorhabditis elegans *orthologues of expressed sequence tags (ESTs) unique to either the ensheathed (= L3) (a) or exsheathed (= xL3) (b) larval stage of *Haemonchus contortus *(see Methods); Representing L3: *ced-10 *was linked to axon guidance; *krs-1 *to lysine biosynthesis; *pas-4 *and *pas-5 *to the proteasome system; *cul-3*, *elc-1 *and *smo-1 *to ubiquitin; F21D5.7 to protein export; *rpl-5*, *rps-14*, *rpl-24.1*, *rpl-32 *and Y37E3.8a to ribosome. Representing xL3: F57B10.3a was linked to glycolysis; *ncs-1 *to olfactory signal transduction; *cpr-6 *to antigen processing and presentation.

**Figure 3:**
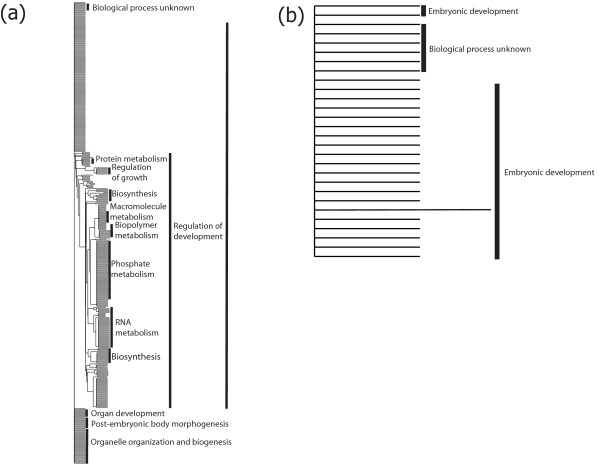
**Classification of interacting genes**. Categorization of genes predicted to interact with *Caenorhabditis elegans *orthologues of expressed sequence tags (ESTs) unique to each ensheathed (= L3) (a) and exsheathed third-stage larvae (= xL3) (b) of *Haemonchus contortus *(see Methods), clustered according to 'biological process' (Gene Ontology). The Gene Ontology hierarchies for individual clusters are given where known.

Twenty-one *C. elegans *orthologues (i.e. genes *elc-1*, *rps-22*, *sod-2*, *rps-14*, *rpl-24.1*, *rpl-32*, *pas-4*, *ced-10*, T10B11.2, *krs-1*, *rpl-31*, *pas-5*, *cul-3*, F21D5.7, *rps-27*, *smo-1*, *rps-11*, Y37E3.8a, *rpl-5*, *col-97 *and F57A8.2b) unique to L3 (cf. Additional file 5) were predicted to interact directly with a total of 535 other genes (range: 1-227, see Figure 2a and Additional file 7). In contrast, seven *C. elegans *orthologues (i.e. *ncbp-2*, F57B10.3a, *vab-9*, *ncs-1*, *cpr-6*, Y52B11A.2a and *nhr-80*) unique to xL3 (see Additional file 6) were inferred to interact with 45 other genes (range 1-27; see Figure 2b and Additional file 7). The functional classification of the *C. elegans *orthologues of molecules which were unique to L3 revealed one predominant group linked to 'embryonic development' (within 'biological process'); this cluster contained molecules associated with 'regulation of development' and other pathways, including 'positive regulation of growth', 'RNA metabolism' and 'biosynthesis' (Figure 3a; Additional file 7). Orthologues unique to xL3 also revealed one main cluster associated with 'embryonic development' (within 'biological process') (Figure 2b; Additional file 7).

## Discussion

In *H. contortus*, the transition to parasitism was proposed to be accompanied by differences in the levels of transcription of particular genes that may facilitate the invasion of the host and the evasion of the immune response [12]. Next-generation sequencing technology was used here to investigate the transcriptome of the *H. contortus *L3 before and after the exsheathment process *in vitro*. Interestingly, ~75% of the sequences determined did not have orthologues/homologues in any other organism for which sequence data are available. However, annotation was enhanced through the mining of genomic data available for *H. contortus *(http://www.sanger.ac.uk; August 2008), ultimately increasing the percentage of annotated sequences to ~50% for the L3 and xL3 datasets (see Results section). The likely explanation for this result is technical and would appear to relate to a 3'-bias in sequence reads [27], thus affecting the prediction of open reading frames (ORFs) and, thus, the identification of orthologues/homologues. In the absence of the complete genome sequence for *H. contortus*, genomic mining provided the sole option for maximizing the mapping and subsequent annotation of sequences.

The integrated approach of using both transcriptomic and available genomic data allowed the annotation of a total of 11,302 sequences, of which 5,117 matched unique *C. elegans *orthologues. Of the 3,010 unique InterPro domains identified, the most represented in both L3 and xL3 were the 'transthyretin-like' (InterPro: IPR001534), 'calcium-binding EF-hand' (IPR002048), 'NAD(P)-binding' (IPR016040) and 'sperm-coating protein (SCP)-like extracellular' (IPR014044) motifs (Additional file 1). Most 'transthyretin' proteins identified to date are conserved (across large evolutionary distances) [28] and have been recognized as enzymes of the purine catabolism that catalyse the conversion of 5-hydroxyisourate (HIU) to OHCU [29,30]. Of the 4,000 'nematode-specific' protein families encoded by 'nematode-restricted' genes, the 'transthyretin-like' proteins (TTLs) [31] represent one of the largest groups [32]. Indeed, members of the TTL family have been detected in plant parasitic nematodes, such as *Xiphinema index, Heterodera glycines*, *Meloidogyne incognita *and *Radophilis similis *[31,33-35], the filarial nematode of humans, *Brugia malayi *[36], and *Ostertagia ostertagi *of ruminants (which is related to *H. contortus*) [37,38]. In *O. ostertagi*, at least 18 *ttl *genes have been identified by database mining; most of these genes are constitutively transcribed from the free-living L3 stage through to adult males and females [38]. In parasitic nematodes, TTLs might play a role as carriers of lipophilic substances or hormones [32,39]. More recently, Jacob and coworkers [31] localized the expression of *ttl *genes to the ventral nerve of *R. similis *(i.e. *Rs-ttl-2*) and to the nervous system and hypodermis of *C. elegans *(gene code R13A5.6; http://www.wormbase.org). These findings, together with the sequence similarity between TTLs and some neuropeptides, suggest that these proteins play a yet uncharacterized role in the nematode nervous system [31]. In *H. contortus*, a TTL has been identified previously using a proteomic approach and shown to be the most immunogenic protein in excretory/secretory products (E/S) of the adult stage [40]. TTLs are also highly abundant in E/S of the canine hookworm, *A. caninum *[41]. These latter two studies propose a central role for these proteins in the parasite-host interplay [40,41], which warrants detailed investigations.

Some host-parasite interactions have been reported to rely on the essential role played by calcium-mediated signalling pathways in protein secretion, motility, cell invasion and/or differentiation [42-44]. These functions are controlled by various, specialized subcellular structures (such as the Golgi apparatus, and some channels and transporters) for the uptake and release of calcium, which acts as a secondary messenger for the activation of calcium-dependent proteins [45], particularly those containing 'EF-hand' domains. In the present study, the large number of sequences (equating to 6.7% overall) with such domains in the L3 and xL3 stages of *H. contortus *might reflect a substantial need for calcium ions in the cascade of developmental events occurring during larval growth, particularly those linked to the maturation of the nervous system, as proposed to function in the *C. elegans *dauer stage [46]. Similarly, the redox reactions catalysed by oxidoreductases with a NAD(P)-binding domain are essential for all developmental processes [47]. The NAD+, a coenzyme found in all living cells, is involved in redox reactions and carries electrons from one reaction to another. In contrast, the main function of NADP+ is as a reducing agent in anabolism, with this coenzyme being involved in key pathways, such as fatty acid synthesis and glucose metabolism [47].

Another relatively abundant group of sequences in both L3 and xL3 encoded key SCP/Tpx-1/Ag5/PR-1/Sc7 proteins (designated SCP/TAPS; Pfam accession number no. PF00188), characterized by one or more SCP-like extracellular domains (InterPro: IPR014044, *p *< 1e-05) [48]. In parasitic nematodes, such proteins are also called *Ancylostoma *secreted proteins or activation-associated proteins (ASPs), mainly because they were originally discovered in hookworms [18,49]. Although the numbers of sequences encoding SCP/TAPS (187 and 207, assembled into three and seven EST clusters, respectively) were similar in L3 and xL3 (cf. Table 3), there were some qualitative differences in transcription between these two stages. Homologues of the molecules designated Hc24 and Hc40, previously identified in excretory/secretory (ES) products of adult *H. contortus *[50,51], were encoded in both of these stages. However, other previously undescribed SCP/TAPS, such as an inferred protein homologue (encoded by *C. elegans *gene F09B9.5) with no known homologues in any other nematode, were encoded only by sequences in xL3 (cf. Table 3). The finding of entirely novel SCP/TAPS supports a previous hypothesis, formulated based on observations from transcriptomic and proteomic analyses, that a broader range of molecules of this group is expressed in *H. contortus *[41]. This finding also suggests a diversified, active and specific involvement of SCP/TAPS in the exsheathment process, establishment of *H. contortus *in its host and/or the parasite-host interplay. Current evidence indicates that the transcriptional profile for *H. contortus *in the transition from L3 to xL3 is quite distinct from that of *A. caninum*, in that there was a massive expansion in SCP/TAPS in the serum-activated L3 (i.e. xL3) in the hookworm [18]. Although there were methodological differences between studies, particularly in relation to the use of normalized cDNA libraries herein, an apparent difference in the diversity of transcripts encoding SCP/TAPS might reflect distinct roles for various types of these molecules in *H. contortus *compared with *A. caninum *during the invasion process in the host. Specifically, L3s of *H. contortus *are ingested by the ruminant host, exsheath in the reticulo-rumen and establish in the abomasum, in which the xL3s start feeding on blood to subsequently develop into dioecious adults [1]. In contrast, infective L3s of *A. caninum *penetrate skin of the canid host, exsheath and then migrate as xL3s *via *the circulatory system and the lung to finally reside as adults in the small intestine; the dioecious hookworm adults attach *via *their buccal capsule to the intestinal mucosa, rupture capillaries and feed on blood [52]. Some xL3s also undergo somatic migration to then encyst in tissues, such as muscle and the mammary gland, and become arrested as L3s [53-55]. Therefore, the added biological complexity in the hookworm life cycle, particularly in the initial phases of invasion and migration as an xL3, compared with the relatively simple gastric infection process for *H. contortus*, supports the current proposal of a very distinct arsenal of SCP/TAPS (both qualitatively and quantitatively) between the two nematodes during their transition to parasitism. Nonetheless, only a direct comparison of transcription between L3 and xL3 for the two parasites using the same or a similar approach as employed herein will allow this hypothesis to be tested conclusively.

**Table 3:** SCP/Tpx-1/Ag5/PR-1/Sc7 (SCP/TAPS) homologues.

	EST code	Length (bp)	Homologue	E-value
L3	Contig875	530	*Ancylostoma caninum Ancylostoma*-secreted protein 1 (ASP-1)	1e - 09
	FJISXER02CFZ04	250	*Haemonchus contortus *Hc24	1e - 22
	FJISXER02CG1I0	263	*Ancylostoma caninum Ancylostoma*-secreted protein 2 (ASP-2)	6e -17

xL3	Contig990	337	*Ancylostoma caninum Ancylostoma*-secreted protein 1 (ASP-1)	2e - 41
	Contig1141	271	*Meloidogyne arenaria *venom allergen-like protein-1 (*Ma*VAP-1).	6e - 09
	Contig2101	431	*Caenorhabditis elegans *hypothetical protein F09B9.5	2e - 28
	Contig2955	256	*Ostertagia ostertagi *activation associated secreted protein ASP-4 (Oo-ASP-4)	9e -16
	Contig3360	315	*Haemonchus contortus *Hc40	3e - 24
	Contig4207	351	*Haemonchus contortus *Hc40	4e - 53
	Contig4530	355	*Ancylostoma ceylanicum Ancylostoma*-secreted protein 1 (ASP-1)	4e - 13
	FJISXER05F8XC0	246	*Ancylostoma caninum Ancylostoma*-secreted protein 5 (ASP-5)	1e - 05
	FJISXER06G7M9K	242	*Meloidogyne arenaria *venom allergen-like protein-1 (*Ma*VAP-1)	4e - 05
	FJISXER06GY7FZ	266	*Haemonchus contortus *Hc40	2e - 17

Expressed sequence tags (ESTs) inferred to encode SCP/TAPS proteins in the ensheathed (= L3) or exsheathed (= xL3) third larval stage of *Haemonchus contortus*.

The *in silico *subtraction approach employed in the present study identified 560 and 421 sequences that were specific to *H. contortus *L3 and xL3, respectively. The specificity of a subset of these transcripts was unequivocally verified by rtPCR-coupled sequencing (see Results section). Most of the subset of 'L3-specific' molecules with known orthologues in *C. elegans *encoded ribosomal proteins (n = 32), collagens (n = 10) and elongation factors (n = 11) (cf. Table 2; Additional file 4). In nematodes, the synthesis of collagens has been observed to increase significantly prior to a moult [56], whereas ribosomal proteins have been reported to participate in various cellular processes besides protein biosynthesis. For instance, they can act as components of the translation apparatus and also regulate cell proliferation and apoptosis [57]. Indeed, genetic interaction networking for *C. elegans *orthologues of *H. contortus *L3-specific transcripts predicted a number of genes coding for ribosomal proteins (i.e. *rps-11*, *rps-14*, *rps-22*, *rps-27*, *rpl-5 *and *rpl-24.1*) to interact directly with a GTPase (i.e. *ced-10*), which is required for phagocytosis during programmed cell death and for migration of the distal tip cells of the somatic gonad [58] (see Figure 2a; Additional file 7). In addition, the protein CED-10 has been demonstrated to be essential in the cascade of events which leads to the development of the nervous system in the growing and developing larva, by guiding the migrating cells and axonal growth cones to their final position in the body [59]. A direct interaction was also predicted between *ced-10 *and another *C. elegans *orthologue of an L3-specific transcript (i.e. *pas-4*) encoding a proteasome alpha-type seven subunit of the core 20S proteasome subcomplex (see Figure 2a; Additional file 7;(http://www.wormbase.org) involved in the 'ubiquitin proteasome system' (UPS) [60]. Loss-of-function in this system is known to be the primary cause or secondary consequence of pathological conditions of the nervous system (e.g., [61]). Moreover, gene silencing of *pas-4 *in *C. elegans *has been shown to result in a wide variety of defects, including embryonic and larval lethality, sterility, abnormal locomotion, slow growth, and abnormal transgene expression and subcellular localization [60]  http:// http://www.wormbase.org. Here, both *ced-10 *and *pas-4 *were predicted herein to interact with a *C. elegans *orthologue of SUMO (i.e. *smo-1*, Figure 2a; Additional file 7). SUMO is a small ubiquitin-like moiety that, when attached to protein substrates, regulates subcellular localization and activity [62,63]. The loss of function of *smo-1 *results in developmental defects of the nervous and reproductive systems as well as embryonic or larval lethality [62,63]. Based on the genetic interaction networks predicted for orthologues of *H. contortus *L3-specific molecules, it could be proposed that axon guidance and synapse formation, which are primarily controlled by UPS, through the regulation of protein turnover at the growth cone and the synapse [61], are crucial in the biology of the L3 of this parasitic nematode. This statement is supported by the fact that the exsheathment process is triggered by gaseous CO_2_, detected by chemosensory neurons of amphids, located in the anterior end of the L3 [7,8], ultimately leading to the secretion of the neurotransmitter noradrenaline [1,3].

Of the *H. contortus *xL3-specific transcripts, those predicted to be involved in mechanisms regulating chemotaxis during larval development were also highly represented. For example, the *C. elegans *orthologue *ncs-1 *encodes a neuronal calcium sensor protein, whose expression in *C. elegans *is primarily associated with two sensory organs, the head amphids and tail phasmids [64]. This orthologue was predicted to interact with 11 distinct genes involved in axon guidance, neuron projection, embryonic and/or larval development (see Figure 2b; Additional file 7). However, the largest number of genetic interactions was predicted for a *C. elegans *orthologue of a *H. contortus *xL3-specific transcript encoding a subunit of the nuclear cap-binding complex (i.e. *ncbp-2*, Figure 2b; Additional file 7). This complex includes RNA-binding proteins that bind to the 5'-cap within the nucleus; when RNA is exported to the cytoplasm, the nuclear cap-binding protein complex is replaced by cytoplasmic cap-binding complex [65]. Although *ncbp-2 *has been demonstrated to play key roles in mRNA decay, embryonic/larval development, reproduction and vulval morphogenesis [66], its precise role in the biology of the developing larva is still unclear.

The largest number of *C. elegans *orthologues of *H. contortus *xL3-specific transcripts encoded peptidases and other enzymes of the amino acid catabolism (cf. Additional files 4 and 6). A similar spectrum of proteases and other molecules linked to catalytic activity have been shown also to be highly represented in the transcriptome of the serum-activated xL3 stage of *A. caninum *by comparison to its L3 [18]. This finding, for two haematophagous bursate nematodes with differing biologies, is likely to reflect the key roles that these molecules play in host tissue invasion, degradation and/or digestion [1,18]. In particular, a cysteine protease (i.e. *cpr-6*) was amongst the *C. elegans *orthologues of xL3-specific molecules in *H. contortus *(see Additional file 6). This finding is supported by previous evidence, showing that cysteine proteases play a crucial role in the catabolism of globin peptides by the cleavage of haemoglobin in blood-feeding nematodes (e.g., *A. caninum *and *Necator americanus*) [67-70]. Given this role, these proteases have been considered as promising candidates for developing recombinant vaccines against *H. contortus *as well as hookworms [71,72]. Typically, these proteases include a 'globin domain' that protects the haeme iron from rapid oxidation and regulates oxygen homoeostasis [73] in the gut of the host, which is characterized by a reduced oxygen tension [74]. Indeed, this domain (represented by InterPro codes IPR000971, IPR012292 and IPR009050, cf. Figure 1a; Additional file 1) was identified as the most abundant motif amongst the xL3-specific molecules. In *H. contortus*, transcripts encoding cathepsin B cysteine protease-like (CBL) proteins have been reported to be most abundant (~16%) in the intestine of the adult female [75]. In addition, a comparison of *H. contortus *isolates from Europe and North America has revealed a minor to moderate nucleotide diversity in *cbl *genes, which has been proposed to reflect antigenic variation among CBLs [75]. Clearly, *C. elegans *provides a useful surrogate system [76] to express *H. contortus *cysteine-proteases and to assess their function(s).

## Conclusion

The present study has provided new and exciting insights into the molecular biology of the L3 stage of *H. contortus *and has elucidated transcriptional alterations taking place during the transition from the free-living to the parasitic stage of this nematode. Although approximately half of the sequences generated by 454 sequencing remains uncharacterised, as a consequence of the absence of complete genomic information for this parasite, future sequencing efforts using, for example, Illumina technology, should provide the efficiency and depth of coverage required to define the complete transcriptomes of all developmental stages and various tissues as well as the genome of *H. contortus*. The determination of the genome sequence of *H. contortus *has major potential to accelerate large-scale studies of genes and gene products involved in nematode development and reproduction, parasite-host interactions and the disease caused by the parasite. Importantly, comprehensive genomic and transcriptomic data will also underpin future proteomic and metabolomic studies of *H. contortus*. Such an integrated approach should lead to important conceptual advances in our understanding of various aspects of nematode biology and should have major implications, in the medium to long term, for the development of novel strategies for parasite intervention, resulting in biotechnological outcomes (such as drugs and diagnostic tests). For instance, future work could focus on defining a spectrum of key molecules involved in pathways linked to the development of the nervous system in different stages of *H. contortus *and assessing their potential as drug targets. Moreover, determining the structure and function of SCP/TAPS homologues/orthologues could establish their role(s) in the invasion of and establishment of this parasite in the host animal, providing the prospect of interfering with the host-parasite relationship. Although the present study focused on *H. contortus*, the research findings and the integrated technological approach employed should find broad applicability to other parasitic nematode of major global importance.

## Methods

### Parasite material

L3s of *H. contortus *("Haecon5 strain") were cultured [77] from the faeces from an experimental sheep with a monospecific infection of this strain using a protocol described previously [78]. Animal ethics approval (AEC no. 0707528) was given by The University of Melbourne, and the care and maintenance of sheep followed this institution's guidelines. The L3s were sieved (mesh size: 20 μm), washed extensively in sterile water and then stored at 10°C for 18 days. L3s (n = 500,000) were exsheathed [10], consistently achieving an exsheathment rate of 99%. Exsheathment using CO_2_* in vitro *[10] is recognised to represent the process *in vivo *within the host animal [10,11]. L3s and xL3s were each snap frozen in liquid nitrogen and then stored at -70°C until use.

### Library construction and next-generation sequencing

For each L3 and xL3 of *H. contortus*, a normalized cDNA library was constructed (Eurofins MWG Operon, Ebersberg, Germany). In brief, total RNA was extracted from either L3 or xL3 using the mirVana isolation kit (Ambion). The integrity of each RNA sample was verified using a Bioanalyzer 2100 (Agilent Technologies), and the yield determined spectrophotometrically (ND-1000 UV-VIS v.3.2.1, NanoDrop Technologies). RNA was then treated with *DNA*se I (DNAfree, Ambion) and polyA+ RNA purified from ~120 ng of total RNA. For each library constructed, first-strand cDNA was synthesized using an oligo(dT)-adapter primer, and the second-strand was produced using a random (N)_6_-adapter primer. The resultant double-stranded cDNA was amplified (21 cycles) by Long and Accurate PCR (LA-PCR) [79] and an aliquot (~200 ng) examined electrophoretically. Normalization was conducted using one cycle of denaturation and reassociation of the cDNA, followed by purification of the reassociated double-stranded cDNA on a hydroxylapatite column. The normalized cDNA (500-700 bases) was then amplified using 9 cycles of LA-PCR and sequenced in a Genome Sequencer™ (GS) FLX Instrument (Roche Diagnostics) employing a standard protocol [19].

### Bioinformatic analyses

ESTs determined from the L3 and xL3 libraries were subjected to separate bioinformatic analyses. For each stage, all ESTs were pre-processed (SeqClean [80]; RepeatMasker [81]), aligned and then clustered using the Contig Assembly Program v.3 (CAP3) [82], employing a minimum sequence overlap of 30 bases and an identity threshold of 95% (for the removal of the flanking adapter sequences), and assembled. The small number of sequences (n = 55; 0.24% of 23,245 contigs) with a perfect match to those available for *Ovis aries *[GenBank: GI:3288836-GI:280977729; e-value < 1e -15] were eliminated from each dataset. Both unassembled and assembled EST datasets were compared, at the nucleotide level using BLASTn (e-value < 1e -05), with genomic sequence data publicly available for *H. contortus *(http://www.sanger.ac.uk; 21st August 2008). Contigs and singletons in each EST dataset, and the corresponding genomic sequence(s), were subjected to BLASTx (NCBI: http://www.ncbi.nlm.nih.gov) and BLASTn (EMBL-EBI Parasite Genome BLAST Server: http://www.ebi.ac.uk) searches to identify putative orthologues/homologues in *C. elegans*, other nematodes, and organisms other than nematodes (e-value of < 1e-05). WormBase (http://www.wormbase.org) was interrogated for relevant information on *C. elegans *orthologues/homologues, including RNAi phenotypes as well as transcriptomic, proteomic and interactomic data. Orthologues/homologues predicted from individual ESTs were always consistent with those inferred from genomic sequences (not shown). ESTs with no match to any sequences in the NCBI and/or EMBL-EBI databases were subjected to BLASTn analysis against the genomic data for *H. contortus *to increase the likelihood of identifying orthologues/homologues in currently available databases.

Following the conceptual translation of contigs and singletons into peptides using ESTScan [83], functional annotation was performed by GO using BLAST2GO [84]. Domains/motifs within translated peptides were identified *via *InterProScan [85] and linked to pathways in *C. elegans *using KOBAS [86]. The open reading frames (ORFs) inferred from sequences with orthologues in *C. elegans *were also subjected to 'secretome analysis' using the program SignalP v.2.0 (available at http://www.cbs.dtu.dk/services/SignalP/), employing both the neural network and hidden Markov models to predict signal peptides and/or anchors [87-89]. In addition, transmembrane domains were inferred using the program TMHMM (http://www.cbs.dtu.dk/services/TMHMM/; [90-92]).

### Identification of transcripts unique to either L3 or xL3 by *in silico *subtraction, and verification of specificity by PCR-coupled sequencing

In order to identify transcripts unique to each stage, the L3 and xL3 datasets were subtracted from one another (in both directions) using *in silico *subtraction (at both the nucleotide and the amino acid levels) employing the BLASTn and tBLASTx algorithms, set at a stringent cut-off (e-value of < 1e-15). Subsets of molecules (sequences) that were present in either L3 (n = 10) or xL3 (n = 10) or in both stages (n = 10) were selected for subsequent experimental verification of specificity by rtPCR. Forward and reverse primers designed to selected contig sequences and corresponding genomic sequences (available at http://www.sanger.ac.uk) (Additional file 8), ensuring that at least one intronic region in the genomic sequence was included (per sequence) to enable the detection by rtPCR of any possible residual genomic DNA in the RNA used. The specificity of each primer designed was evaluated *in silico *by BLASTn analysis against all presently available databases, including that containing *H. contortus *genomic data. The rtPCR was carried out as described previously [93] with the following modifications: (i) the cycling conditions were: 95°C, 2 min (initial denaturation) followed by 35 cycles of 95°C for 1 min (denaturation), 55°C for 1 min (annealing) and 72°C for 1 min (extension), and a final extension of 72°C for 5 min; (ii) in addition to part of the β-tubulin (250 bp) gene being used as a positive control, a portion of the elongation factor-1α (216 bp) gene was included for the amplification (cf. Additional file 8). Negative control reactions using template RNA from L3 and xL3 were also included. All amplicons produced were sequenced [94] to demonstrate unequivocally that they represented the correct sequence. The Fisher's exact test was used to confirm that the number of molecules selected from each dataset (obtained following *in silico *subtraction and containing sequences unique to either L3 or xL3) for the verification of differential transcription by rtPCR was representative statistically (*p *< 0.001).

### Probabilistic functional gene networking

The method developed by Zhong and Sternberg [95] was used to predict the interaction networks among *C. elegans *orthologues of molecules transcribed in either L3 or xL3. Data regarding interactions, phenotypes, expression and GO for selected *C. elegans *gene orthologues/homologues, also incorporating data from *Drosophila melanogaster *(vinegar fly), *Saccharomyces cerevisiae *(yeast), *Mus musculus *(mouse) and *Homo sapiens *(human), were integrated using a naïve Bayesian model to predict genetic interactions among *C. elegans *genes using the recommended, stringent cut-off value of 4.6 [94,95]. The predicted networks resulting from the analyses were saved in a graphic display file (gdf) format, examined using the graph exploration system available at http://graphexploration.cond.org/ and drawn using Adobe Illustrator CS2 (Adobe Systems Inc.). The genes predicted to interact with selected transcripts unique to L3 and xL3 were also classified according to the 'Biological process Gene Ontology annotations of their interacting partners' using the PROSTIDIN website (http://crfb.univ-mrs.fr/webdistin/; [96]).

## Authors' contributions

CC performed the bioinformatic analyses, analysed the results and drafted the manuscript, BEC, NDY, ARJ and RSH also participated in the bioinformatic analyses, PJAP and JLZ provided the parasite material, WZ and BAM assisted in the probabilistic genetic interaction network predictions, AL and PWS contributed to the drafting of the manuscript. RBG conceived and designed the study, coordinated and supervised the project and drafted the manuscript with CC. All authors read and approved the final manuscript.

## Supplementary Material

Additional file 1 **Protein motifs**. InterPro domains predicted peptide sequences encoded in either the ensheathed (= L3) or exsheathed (= xL3) third larval stage of *Haemonchus contortus*. Click here for file

Additional file 2 **Gene ontology (GO)**. Terms (i.e. 'biological process', 'cellular component' and/or 'molecular function') representing proteins inferred to be encoded by either the ensheathed (= L3) or exsheathed (= xL3) third larval stage of *Haemonchus contortus*. Click here for file

Additional file 3 **Prediction of biological pathways**. Pathways predicted for molecules inferred to be encoded in either the ensheathed (= L3) or exsheathed (= xL3) third larval stage of *Haemonchus contortus*. Click here for file

Additional file 4 **List of *Caenorhabditis elegans *orthologues**. *C. elegans *orthologues of expressed sequence tags (ESTs) and genome survey sequences (GSS) representing either the ensheathed (= L3) or exsheathed (= xL3) third larval stage of *Haemonchus contortus*. Click here for file

Additional file 5 **Expressed sequence tags (ESTs) unique to the ensheathed third larval stage (L3)**. Bioinformatic characterisation of ESTs encoding molecules uniquely transcribed in the L3 of *Haemonchus contortus *with orthologues in *Caenorhabditis elegans *and other parasitic nematodes. Click here for file

Additional file 6 **Expressed sequence tags (ESTs) unique to the exsheathed third larval stage (xL3)**. Bioinformatic characterisation of ESTs encoding molecules uniquely transcribed in the xL3 of *Haemonchus contortus *with orthologues in *Caenorhabditis elegans *and other parasitic nematodes. Click here for file

Additional file 7 **Probabilistic genetic interaction network predictions**. A list of *Caenorhabditis elegans *orthologues of expressed sequence tags (ESTs) unique to either the ensheathed (= L3) or exsheathed (= xL3) third larval stage of *Haemonchus contortus *for which probabilistic genetic interaction networks were predicted. Interacting genes are listed according to decreased cut-off scores (see Methods). Click here for file

Additional file 8 **Reverse-transcription polymerase chain reaction (rtPCR)**. The sequences of oligonucleotide primers used in rtPCR (see Methods). Click here for file
