# Supplementary material for: Differences in transcription between free-living and CO2-activated third-stage larvae of Haemonchus contortus
Source: BMC Genomics. 2010 Apr 27;11:266. doi: 10.1186/1471-2164-11-266 (PMC2880303; doi:10.1186/1471-2164-11-266)
Supplement: Additional file 5 — Expressed sequence tags (ESTs) unique to the ensheathed third larval stage (L3). Bioinformatic characterisation of ESTs encoding molecules uniquely transcribed in the L3 of Haemonchus contortus with orthologues in Caenorhabditis elegans and other parasitic nematodes. [file 1471-2164-11-266-S5.DOC]

**Additional file 5** – **Expressed sequence tags (ESTs) unique to the ensheathed third larval stage (L3).** Bioinformatic characterisation of ESTs encoding molecules uniquely transcribed in the L3 of *Haemonchus contortus* with orthologues in *Caenorhabditis elegans* and other parasitic nematodes

| **EST code** | **Size (bp)** | ***In silico* peptide analysis^a^** | **Description of *C. elegans* homologue (gene code, *gene name*)** | **RNAi phenotypes^b^** | **InterProScan analysis** | **KOBAS analysis** | **Other strongylid nematodes [non-strongylid nematodes] ^c^** |
| --- | --- | --- | --- | --- | --- | --- | --- |
| *Contigs* |  |  |  |  |  |  |  |
| Contig115 | 284 | S/0 | CEll Death abnormality family member ([C09G12.8](http://www.wormbase.org/db/seq/sequence?name=C09G12.8;class=Gene_name), *ced-10*) | Distal tip cell migration abnormal | Ras small GTPase, Rho type | VEGF signaling pathway;  B cell receptor signaling pathway; Natural killer cell mediated cytotoxicity; Regulation of actin cytoskeleton; Leukocyte transendothelial migration; Axon guidance; Focal adhesion; Renal cell carcinoma; Epithelial cell signaling in *Helicobacter pylori* infection; MAPK signaling pathway; Fc epsilon RI signaling pathway; Pancreatic cancer; Colorectal cancer; Toll-like receptor signaling pathway; Wnt signaling pathway; GTP-binding proteins | *Lsi, Mha, Min, Oos, Ovo, Ptr* [*Mha*] |
| Contig1442 | 487 | S/0 | APurinic/apyrimidinic endoNuclease family member ([T05H10.2](http://www.wormbase.org/db/seq/sequence?name=T05H10.2;class=Gene_name), *apn-1*) | None |  | None | *Tvi* |
| Contig1565 | 332 | S/0 | Ceramide kinase (T10B11.2) | None |  | None |  |
| Contig1880 | 375 | S/0 | Signal peptidase 1 (Y54E10BR.5) | Sck, Ste | Peptidase S24, S26A and S26B, C-terminal | Peptidases; Protein export | *Gro, Hgl, Mha, Ppa, Sst, Xin* [*Bma, Cbr*] |
| Contig2599 | 461 | S/0 | Cytosine deaminase FCY1 and related enzyme (Y48A6B.7) | None |  | Purine metabolism | *Oos, Tci [Cbr]* |
| Contig3315 | 235 | S/0 | tRNA Synthetase family member ([T02G5.9](http://www.wormbase.org/db/seq/sequence?name=T02G5.9;class=Gene_name), *krs-1*) | Lva, Gro, small, Bmd, Ste, transgene expression increased | Aminoacyl-tRNA synthetase, class II (D, K and N) | Lysine biosynthesis; Aminoacyl-tRNA biosynthesis | *[Ppa]* |
| Contig3401 | 534 | S/0 | Actin regulatory protein (K06A4.3) | None | Gelsolin region | Regulation of actin cytoskeleton  Cytoskeleton proteins | *[Ppa]* |
| Contig4525 | 425 | S/0 | Ribosomal Protein, Large subunit family member ([W09C5.6](http://www.wormbase.org/db/seq/sequence?name=W09C5.6;class=Gene_name), *rpl-31*) | Emb, Ste, Pvl, pleiotropic defects severe early emb |  | None | *Min* |
| Contig4627 | 248 | S/0 | Proteasome Alpha Subunit family member ([F25H2.9](http://www.wormbase.org/db/seq/sequence?name=F25H2.9;class=Gene_name), *pas-5*) | Emb, passage through meiosis abnormal early emb, Let, Lva, Unc | Proteasome alpha-subunit, conserved site | Peptidases  Proteasome | *Ppa, Ptr, Xin* [*Asu, Wba*] |
| Contig5843 | 290 | S/0 | CULlin family member ([Y108G3AL.1](http://www.wormbase.org/db/seq/sequence?name=Y108G3AL.1;class=Gene_name), *cul-3*) | Emb, protein subcellular localization abnormal, protein expression increased, transgene expression increased, pronuclear migration abnormal early emb | Cullin, conserved site | Ubiquitin enzymes | *Gpa, Gro, Hgl, Mch, Mha, Mja, Mpa, Nam, Nbr, Sra* [*Cbr*, *Wba*] |
| *Singletons* |  |  |  |  |  |  |  |
| FJISXER02CBJZS | 252 | S/0 | Acetylcholine receptor (T01H10.6, *lgc-17*) | None | Neurotransmitter-gated ion-channel | None |  |
| FJISXER03D2EHA | 269 | S/0 | Signal recognition particle (F21D5.7) | Lva, Ste | Signal recognition particle, SRP54 subunit, M-domain | Protein export | *Sra, Sst* |
| FJISXER02BWQSA | 257 | S/0 | Temporarily Assigned Gene name family member ([B0228.4](http://www.wormbase.org/db/seq/sequence?name=B0228.4;class=Gene_name), *tag-308*) | None | Copine | Glycolysis / Gluconeogenesis | *Min* [*Bma, Wba*] |
| FJISXER02CKTEC | 294 | S/0 | Predicted RNA-binding protein (Y82E9BR.18) | None |  | None | *Sst* |
| FJISXER02BH3KK | 269 | S/0 | Ribosomal Protein, Small subunit family member ([F56E10.4](http://www.wormbase.org/db/seq/sequence?name=F56E10.4;class=Gene_name), *rps-27*) | Lva, Emb, Gro, pleiotropic defects severe early emb |  | None | *Hgl, Nbr, Ppa, Ptr, Pvu, Rsi* [*Asu*] |
| FJISXER02B12NI | 274 | S/0 | Carboxylesterase (F15A8.6) | None |  | None | *Cbr, Pvu* |
| FJISXER02BHA9A | 283 | S/0 | Sumo (ubiquitin-related) homolog protein 1 ([K12C11.2](http://www.wormbase.org/db/seq/sequence?name=K12C11.2;class=Gene_name), *smo-1*) | Mvl, Emb, Let, Pvl, ectopic expression transgene, blistered, reduced brood size, Gro, Dpy, Ste | Ubiquitin | None | *Aca, Cbr, Nam, Oos, Ppa, Tci* [*Asu*] |
| FJISXER02BHA9S | 261 | S/0 | Ribosomal Protein, Small subunit family member ([F40F11.1](http://www.wormbase.org/db/seq/sequence?name=F40F11.1;class=Gene_name), *rps-11*) | Emb, pleiotropic defects severe early emb, Sck, Ste, Lva, Age, |  | None | *Cbr, Gpa, Gro, Ppa, Ppe, Ptr, Rsi, Xin* |
| FJISXER03DCV5H | 253 | S/0 | Non ribosomal peptide synthetase/alpha aminoadipase reductase (C41A3.1) | None |  | Transporters | *Aca* |
| FJISXER02CB5AT | 313 | S/0 | Collagen (ZK1010.7, *col-97*) | Bmd, Dpy |  | None | *Ptr* |
| FJISXER02BHXAB | 250 | S/0 | Ubiquitin C-terminal Hydrolase (family 1) family member ([F46E10.8](http://www.wormbase.org/db/seq/sequence?name=F46E10.8;class=Gene_name), *ubh-1*) | None |  | Parkinson's disease;  Peptidases | *Ppa* |
| FJISXER02BHECG | 243 | S/0 | Ribosomal Protein, Large subunit family member ([F54C9.5](http://www.wormbase.org/db/seq/sequence?name=F54C9.5;class=Gene_name), *rpl-5*) | Gro, Dpy, Ste, Pvl, Pch, Emb, pleiotropic defects severe early emb, Lva | Ribosomal protein L18/L5 | Ribosome | *Min, Ptr, Sra, Tsp, Xin* [*Asu*] |
| FJISXER02BHBZL | 254 | S/0 | 60S ribosomal protein (Y37E3.8a) | Emb, pleiotropic defects severe early emb |  | Ribosome | *Hgl, Ppa, Rsi* [*Asu*] |
| FJISXER02BHBGH | 255 | S/0 | Elongation factor 1-beta/1-delta 2 ([Y41E3.10](http://www.wormbase.org/db/seq/sequence?name=Y41E3.10;class=Gene_name)) | Lva, Gro | Translation elongation factor EF1ß/ribosomal protein S6 | Translation factors | *Gpa, Hgl, Mar, Sra, Xin* [*Asu, Bma, Wba*] |
| FJISXER02BHHQG | 255 | S/0 | ELongin C family member ([Y82E9BR.15](http://www.wormbase.org/db/seq/sequence?name=Y82E9BR.15;class=Gene_name), *elc-1*) | Emb, complex phenotype early emb, larval arrest | SKP1 component, POZ | Ubiquitin enzymes;  Ubiquitin mediated proteolysis;  Renal cell carcinoma | *Ace, Gro, Mja, Xin* [*Asu*] |
| FJISXER02BHEYT | 240 | S/0 | Ribosomal Protein, Small subunit family member ([F53A3.3](http://www.wormbase.org/db/seq/sequence?name=F53A3.3;class=Gene_name), *rps-22*) | Gro, Sck, Pch, Age, Lva, Emb, Ste, pleiotropic defects severe early emb, developmental delay postembryonic |  | None | *Aca, Ace, Hgl, Mpa, Nbr, Ppa, Sra, Sst,* [*Asu, Tca, Wba*] |
| FJISXER02BHKWR | 257 | S/0 | SOD (superoxide dismutase) family member ([F10D11.1](http://www.wormbase.org/db/seq/sequence?name=F10D11.1;class=Gene_name), *sod-2*) | None |  | None | *Mch, Mha, Oos, Ovo, Ppa, Tvu* |
| FJISXER02BHKPO | 254 | S/0 | Ribosomal Protein, Small subunit family member ([F37C12.9](http://www.wormbase.org/db/seq/sequence?name=F37C12.9;class=Gene_name), *rps-14*) | Emb, Ste, Lva, pleiotropic defects severe early emb, Sck, Gro, reduced brood size | Ribosomal protein S11 | Ribosome | *Ace, Hgl, Nbr, Ppa, Ptr, Tci* [*Asu*] |
| FJISXER02BHKJ5 | 260 | S/0 | Ribosomal Protein, Large subunit family member ([D1007.12](http://www.wormbase.org/db/seq/sequence?name=D1007.12;class=Gene_name), *rpl-24.1*) | Lva, Ste, Emb, pleiotropic defects severe early emb | Ribosomal protein L24e | Ribosome | *Hgl, Hsc, Lsi, Mha, Xin* [*Wba*] |
| FJISXER02BHKHQ | 236 | S/0 | Ribosomal Protein, Large subunit family member ([T24B8.1](http://www.wormbase.org/db/seq/sequence?name=T24B8.1;class=Gene_name), *rpl-32*) | Ste, Lva | Ribosomal protein L32e | Ribosome | *Gpa, Gro, Hgl, Mha, Ppa* [*Asu, Bma*] |
| FJISXER02BHQU9 | 240 | S/0 | Triose Phosphate Isomerase family member ([Y17G7B.7](http://www.wormbase.org/db/seq/sequence?name=Y17G7B.7;class=Gene_name), *tpi-1*) | Age | Aldolase-type TIM barrel | Glycolysis / Gluconeogenesis;  Carbon fixation;  Fructose and mannose metabolism;  Inositol metabolism | *Cbr* |
| FJISXER02BHNOW | 248 | S/0 | Proteasome Alpha Subunit family member ([C36B1.4](http://www.wormbase.org/db/seq/sequence?name=C36B1.4;class=Gene_name), *pas-4*) | Emb, passage through meiosis abnormal early emb, Gro, Lvl, Lva, Unc, transgene expression increased | 20S proteasome, A and B subunits | Proteasome;  Peptidases |  |
| FJISXER03CXLHD | 245 | S/0 | Predicted membrane protein (F57A8.2b) | None | Hrf1 | None | *Aca* |
| FJISXER02CMRST | 272 | S/0 | Unnamed protein (B0280.7) | None |  | None |  |

^a^Abbreviations used in proteomic analysis: Non-secretory protein (Q), secretory protein (S)/ predicted number of transmembrane domains/ predominat cellular location: extracellular (Ex); nuclear (Nu), plasma membrane (Pl), cytoplasm (Cy).

^b^Abbreviations of RNAi phenotypes (alphabetical): lifespan abnormal (Age), body morphology defect (Bmd), dumpy (Dpy) egg laying defective (Egl), embryonic lethal (Emb), slow growth (Gro), larval lethal (Let), larval arrest (Lva), multivulva (Mvl), protruding vulva (Pvl), patchy coloration (Pch), sick (Sck), sterile (Ste), uncoordinated (Unc).

^c^Abbreviations of nematode species (alphabetical): *Ancylostoma caninum (Aca), Ancylostoma ceylanicum (Ace),* *Ascaris suum (Asu), Brugia malayi (Bma), Caenorhabditis briggsae (Cbr), Globodera pallida (Gpa), Globodera rostochiensis (Gro), Heterodera glycines (Hgl), Heterodera schacthii (Hsc), Litomosoides sigmodontis (Lsi), Meloidogyne arenaria (Mar), Meloidogyne chitwoodi (Mch), Meloidogyne hapla (Mha), Meloidogyne incognita (Min), Meloidogyne javanica (Mja), Meloidogyne paranaensis (Mpa), Necator americanus (Nam), Nippostrongylus brasiliensis (Nbr), Ostertagia ostertagi (Oos), Onchocerca volvulus (Ovo), Parastrongyloides trichosuri (Ptr), Pratylenchus penetrans (Ppe), Pratylenchus vulnus (Pvu), Pristionchus pacificus (Ppa), Radopholus similis (Rsi), Strongyloides ratti (Sra), Teladorsagia circumcincta (Tci), Toxocara canis (Tca), Trichinella spiralis (Tsp), Trichostrongylus vitrinus (Tvi), Trichuris muris (Tmu), Wuchereria bancrofti (Wba), Xiphinema index (Xin).*
