# Supplementary material for: Differences in transcription between free-living and CO2-activated third-stage larvae of Haemonchus contortus
Source: BMC Genomics. 2010 Apr 27;11:266. doi: 10.1186/1471-2164-11-266 (PMC2880303; doi:10.1186/1471-2164-11-266)
Supplement: Additional file 6 — Expressed sequence tags (ESTs) unique to the exsheathed third larval stage (xL3). Bioinformatic characterisation of ESTs encoding molecules uniquely transcribed in the xL3 of Haemonchus contortus with orthologues in Caenorhabditis elegans and other parasitic nematodes. [file 1471-2164-11-266-S6.DOC]

**Additional file** **6** - **Expressed sequence tags (ESTs) unique to the exsheathed third larval stage (xL3).** Bioinformatic characterisation of ESTs encoding molecules uniquely transcribed in the xL3 of *Haemonchus contortus* with orthologues in *Caenorhabditis elegans* and other parasitic nematodes.

| **EST code** | **Size (bp)** | ***In silico* peptide analysis^a^** | **Description of *C. elegans* homologue (gene code, *gene name*)** | **RNAi phenotypes^b^** | **InterProScan analysis** | **KOBAS analysis** | **Other strongylid nematodes [non-strongylid nematodes] ^c^** |
| --- | --- | --- | --- | --- | --- | --- | --- |
| *Contigs* |  |  |  |  |  |  |  |
| Contig1048 | 538 | S/0 | Zn-finger protein (F22D6.2) |  | Zinc finger, AN1-type | General function prediction only | *Cbr, Ptr* [*Asu*] |
| Contig2500 | 364 | S/0 | Uncharacterized conserved protein, contains RWD domain (T26E3.4) | Gro, Sck |  |  | *Ace* |
| Contig4162 | 411 | S/0 | Nuclear cap-binding protein complex (F26A3.2, *ncbp-2*) | Ste, Lva, Pvl, Age, RNAi resistant, transgene expression increased |  |  | *Aca, Ace, Cbr, Min, Nam, Oos* |
| *Singletons* |  |  |  |  |  |  |  |
| FJISXER06GVQ3D | 268 | S/0 | CLaudin-like in *Caenorhabditis* family member ([C01C10.4](http://www.wormbase.org/db/seq/sequence?name=C01C10.4;class=Gene_name), *clc-5*) |  | Clc-like |  | [*Wba*] |
| FJISXER06G62DM | 265 | S/0 | Peptidyl-alpha-hydroxyglycine alpha-amidating lyase (F21F3.1) |  | NHL repeat; Six-bladed beta-propeller, TolB-like; Soluble quinoprotein glucose dehydrogenase | Other enzymes | *Aca, Ace, Gro, Hsc, Mha, Mja, Min, Nam, Oos* |
| FJISXER05FOB0V | 256 | S/0 | Phosphoglycerate mutase (F57B10.3a) | Egl, Emb, Gro, Lva, Bmd, Unc, Age | BPG-independent PGAM, N-terminal | Glycolysis / Gluconeogenesis | *Cbr, Lsi, Tmu* |
| FJISXER05GCES1 | 267 | S/0 | Unnamed protein (T14G10.3, *ttr-53*) |  | Transthyretin-like |  | *Ppa, Hgl, Oos* [*Dim*] |
| FJISXER05F3UST | 240 | S/0 | Unnamed protein (T09E8.1f) | Ste, distal tip cell migration abnormal |  |  |  |
| FJISXER05FZ6PZ | 278 | S/0 | Variable ABnormal morphology family member ([T22C8.8](http://www.wormbase.org/db/seq/sequence?name=T22C8.8;class=Gene_name), *vab-9*) | Pathogen susceptibility increased | P53-induced protein |  | *Cbr, Min* |
| FJISXER05F5844 | 281 | S/0 | Zn-finger protein (C27H5.3) |  | Zinc finger, RanBP2-type | Purine metabolism | *Aca, Ace, Ppa* |
| FJISXER06HDYID | 238 | S/0 | Serpentine Receptor, class T family member (*srt-61*) |  |  |  |  |
| FJISXER06HMMZ8 | 253 | S/0 | Globin-like protein ORF1 (ZK637.13, *glb-1*) |  | Globin |  | *Ace, Nbr, Oos, Tci* |
| FJISXER06GRI9D | 259 | S/0 | Neuronal Calcium Sensor family member ([C44C1.3](http://www.wormbase.org/db/seq/sequence?name=C44C1.3;class=Gene_name), *ncs-1*) |  | Calcium-binding EF-hand | Olfactory transduction | *Cbr, Gpa, Gro, Mar, Mha, Min, Ovo, Ptr, Sst* |
| FJISXER05FVV8O | 236 | S/0 | Cysteine PRotease related family member ([C25B8.3](http://www.wormbase.org/db/seq/sequence?name=C25B8.3;class=Gene_name), *cpr-6*) |  | Peptidase C1A, cathepsin B; Peptidase C1A, papain; Peptidase, cysteine peptidase active site | Antigen processing and presentation | *Aca, Ppa, Ptr,Tci, Xin* [*Asu*] |
| FJISXER06HLP6K | 256 | S/0 | Uncharacterized conserved protein (Y52B11A.2a) |  | Protein of unknown function UPF0029, N-terminal |  | *Aca* |
| FJISXER06HKO27 | 259 | S/0 | Peptidyl-alpha-hydroxyglycine alpha-amidating lyase F21F3.1 |  |  | Other enzymes | *Aca, Oos* |
| FJISXER05F10DF | 257 | S/0 | Nuclear Hormone Receptor family member ([H10E21.3](http://www.wormbase.org/db/seq/sequence?name=H10E21.3;class=Gene_name), *nhr-80*) | Emb, Ste, Gro, Let, thin, pale, RNA expression abnormal, transgene expression reduced |  |  |  |
| FJISXER06G9VAC | 235 | S/0 | Protein kinase (K03E5.3, *cdk-2*) | Lva, small, Bmd, Unc, Rol, Rup, Mlt | Serine/threonine protein kinase-related | Small cell lung cancer |  |

^a^Abbreviations used in proteomic analysis: Non-secretory protein (Q), secretory protein (S)/ predicted number of transmembrane domains/ predominat cellular location: extracellular (Ex); nuclear (Nu), plasma membrane (Pl), cytoplasm (Cy).

^b^Abbreviations of RNAi phenotypes (alphabetical): lifespan abnormal (Age), body morphology defect (Bmd), egg laying defective (Egl), embryonic lethal (Emb), slow growth (Gro), larval arrest (Lva), molt defect (Mlt), protruding vulva (Pvl), roller (Rol), ruptured through vulva (Rup), sick (Sck), sterile (Ste), uncoordinated (Unc).

^c^Abbreviations of nematode species (alphabetical): *Ancylostoma caninum (Aca), Ancylostoma ceylanicum (Ace),* *Ascaris suum (Asu), Caenorhabditis briggsae (Cbr), Globodera pallida (Gpa), Globodera rostochiensis (Gro), Heterodera glycines (Hgl), Heterodera schacthii (Hsc), Litomosoides sigmodontis (Lsi), Meloidogyne arenaria (Mar), Meloidogyne hapla (Mha), Meloidogyne incognita (Min), Meloidogyne javanica (Mja), Necator americanus (Nam), Nippostrongylus brasiliensis (Nbr), Ostertagia ostertagi (Oos), Parastrongyloides trichosuri (Ptr), Pristionchus pacificus (Ppa), Teladorsagia circumcincta (Tci), Trichuris muris (Tmu), Xiphinema index (Xin).*
