# Supplementary material for: Differences in transcription between free-living and CO2-activated third-stage larvae of Haemonchus contortus
Source: BMC Genomics. 2010 Apr 27;11:266. doi: 10.1186/1471-2164-11-266 (PMC2880303; doi:10.1186/1471-2164-11-266)
Supplement: Additional file 8 — Reverse-transcription polymerase chain reaction (rtPCR). The sequences of oligonucleotide primers used in rtPCR (see Methods). [file 1471-2164-11-266-S8.DOC]

**Additional file 8 – Reverse-transcription polymerase chain reaction (rtPCR).** The sequences of oligonucleotide primers used in rtPCR (see Methods).

| **Contig name** | **Primer** | **Sequence (5’ – 3’)** | **Tm** | **Length** | **Amplicon size (bp)** |
| --- | --- | --- | --- | --- | --- |
| *L3* |  |  |  |  |  |
| Contig2599 | Forward | GATGACAAG GCTTTCCACGAC | 54 | 21 | 173 |
| Contig2599 | Reverse | GAGCGACGA AGAACTGATCC | 54 | 20 |
| Contig1880 | Forward | GAACCTGCATACTTTCGTGGTGA | 55 | 23 | 178 |
| Contig1880 | Reverse | CTTCGTTGTTGTCTCCCTTGGTT | 55 | 23 |
| Contig1442 | Forward | CTTCGTTGTTGTCTCCCTTGGTT | 55 | 23 | 235 |
| Contig1442 | Reverse | GTTATGAGTGTTAGCGGAGCAAGA | 56 | 24 |
| Contig1565 | Forward | AAGGGAATGCGTTGCACTTTAGG | 55 | 23 | 240 |
| Contig1565 | Reverse | ATCGGAGGGGAGTTCATTCTC | 54 | 21 |
| Contig3315 | Forward | TGGGATGCTCTGGAGGATAG | 54 | 20 | 181 |
| Contig3315 | Reverse | GGTGTTCGACGCATTCTCTGA | 54 | 21 |
| Contig3401 | Forward | GCCGAAAAGAAGGGTGGTCAAA | 55 | 22 | 206 |
| Contig3401 | Reverse | GCTAATGCTTTACGTCTTTCGTG | 53 | 23 |
| Contig4525 | Forward | GCCATCAGAGATCCGCAAGTT | 54 | 21 | 173 |
| Contig4525 | Reverse | GTACAGCTTGTTGGGGGAGTC | 56 | 21 |
| Contig4627 | Forward | CAATGAGTAGCGATGTTTCTGACA | 54 | 24 | 230 |
| Contig4627 | Reverse | TGTTCATCGACCTTGCTGAT | 50 | 20 |
| Contig1558 | Forward | CGCGTATATCGTCGTTACCC | 54 | 20 | 190 |
| Contig1558 | Reverse | GGGCGAAATATACAGCGAGA | 52 | 20 |
| Contig1675 | Forward | GCAACGTACCGAGCTCTACC | 56 | 20 | 190 |
| Contig1675 | Reverse | TGATACATCTGAAGAACCCAAGG | 53 | 23 |
| *xL3* |  |  |  |  |  |
| Contig1048 | Forward | AGCAATCTGGAACCGACAGTG | 54 | 21 | 265 |
| Contig1048 | Reverse | CCTCATGCTCCATCGTTTTGTA | 53 | 22 |
| Contig4162 | Forward | GATACCGAAAATGCCGTACGTT | 53 | 22 | 200 |
| Contig4162 | Reverse | TCCTCTCCCGATCTTGCCAGA | 56 | 21 |
| Contig2500 | Forward | CAGCAATCTGGAACCGACAGTG | 57 | 22 | 196 |
| Contig2500 | Reverse | GCAGCTTCACGTTCCTTCTCC | 56 | 21 |
| Contig602 | Forward | GGACGGGAAATGTAGAAGAGG | 54 | 21 | 195 |
| Contig602 | Reverse | GTAACTTCGGTGTTGTCGGTTTC | 55 | 23 |
| Contig799 | Forward | CAGCAATCTGGAACCGACAGTG | 57 | 22 | 234 |
| Contig799 | Reverse | AATTGAAGCGTTCTGTTTGCAGTC | 54 | 24 |
| Contig2995 | Forward | CGGCCAAGACCGTTTAGGTTTT | 55 | 22 | 187 |
| Contig2995 | Reverse | TCAATGCACACTTTAGATACGTTAG | 55 | 21 |
| Contig3693 | Forward | GAAATGGAGCCAGAATCAACCTG | 55 | 23 | 246 |
| Contig3693 | Reverse | CATACACCATATCCGCCCGATA | 55 | 22 |
| Contig3766 | Forward | CCGGCGAAGTGGATTTTCTACA | 55 | 22 | 170 |
| Contig3766 | Reverse | TTAGTGTGCGTAGCCCAACTG | 54 | 21 |
| Contig3803 | Forward | ATCTGGAACCGACAGTCAGC | 54 | 20 | 213 |
| Contig3803 | Reverse | CGATTGGAGCGATTGGAACAAC | 55 | 22 |
| Contig4162 | Forward | CGCGAGCAGATACCGAAAATG | 54 | 21 | 219 |
| Contig4162 | Reverse | AGCCATTCAAATCCTCTCCCG | 54 | 21 |
| *L3 and xL3* |  |  |  |  |  |
| Contig6258 | Forward | GAACAGATCCCGCCTACGTC | 56 | 20 | 199 |
| Contig6258 | Reverse | GCTCCCAAGCCCTTGATGATA | 54 | 21 |
| Contig42 | Forward | AGTGAGTAGCCAAGGCTGGA | 54 | 20 | 184 |
| Contig42 | Reverse | CCATTCGGTCCCAACTCTAAC | 54 | 20 |
| Contig6273 | Forward | GAAGCCTGAACAGCACTGGAT | 54 | 21 | 244 |
| Contig6273 | Reverse | AACACATGGGAACAGCCAACG | 54 | 21 |
| Contig6275 | Forward | AGTTCTTTGCGACGAAAGTATTTGGAA | 55 | 27 | 187 |
| Contig6275 | Reverse | ACTGGATCACAGCCAAGGAC | 54 | 20 |
| Contig28 | Forward | GAATCGAAGCCGGCTAATGTGTAT | 56 | 24 | 210 |
| Contig28 | Reverse | CCTTTTCAGGCGGAGTGTAG | 54 | 20 |
| Contig4 | Forward | CAGATGGCAAACAGAAACGAATATGG | 56 | 26 | 226 |
| Contig4 | Reverse | GTTCAGTTCTCGCCCTTGAGT | 54 | 21 |
| Contig9 | Forward | CGGCCTGCTGGTACCCAACTAA | 59 | 22 | 216 |
| Contig9 | Reverse | GGCTTCGTGTGAACTTTCCCCAAAT | 58 | 25 |
| Contig6280 | Forward | TGGAACCGACAGTGAGTATTTG | 53 | 22 | 166 |
| Contig6280 | Reverse | CATGCAGTCGTTTTGCAAGT | 50 | 20 |
| EF-1α | Forward | GGAAGTTCGAGACAGCCAAG | 59 | 20 | 216 |
| EF-1α | Reverse | CGATAAGCTGCTTCACACCA | 60 | 20 |
| ß-Tubulin | Forward | TTCCCTGGACARCTRAATGCT | 50 to 54 | 21 | 250 |
| ß-Tubulin | Reverse | CGCATGCTCATYCGACGACGG | 58 to 60 | 21 |
